# Dietary Administration of Scallion Extract Effectively Inhibits Colorectal Tumor Growth: Cellular and Molecular Mechanisms in Mice

**DOI:** 10.1371/journal.pone.0044658

**Published:** 2012-09-14

**Authors:** Palanisamy Arulselvan, Chih-Chun Wen, Chun-Wen Lan, Yung-Hsiang Chen, Wen-Chi Wei, Ning-Sun Yang

**Affiliations:** 1 Agricultural Biotechnology Research Center, Academia Sinica, Taipei, Taiwan, Republic of China; 2 Molecular and Biological Agricultural Sciences, Taiwan International Graduate Program, National Chung-Hsing University and Academia Sinica, Taipei, Taiwan, Republic of China; 3 Graduate Institute of Biotechnology and Department of Life Sciences, National Chung-Hsing University, Taichung, Taiwan, Republic of China; 4 Department of Marine Biotechnology and Resources, National Sun Yat-Sen University, Kaohsiung, Taiwan, Republic of China; 5 Institute of Biotechnology, National Taiwan University, Taipei, Taiwan, Republic of China; University of California Irvine, United States of America

## Abstract

Colorectal cancer is a common malignancy and a leading cause of cancer death worldwide. Diet is known to play an important role in the etiology of colon cancer and dietary chemoprevention is receiving increasing attention for prevention and/or alternative treatment of colon cancers. *Allium fistulosum* L., commonly known as scallion, is popularly used as a spice or vegetable worldwide, and as a traditional medicine in Asian cultures for treating a variety of diseases. In this study we evaluated the possible beneficial effects of dietary scallion on chemoprevention of colon cancer using a mouse model of colon carcinoma (CT-26 cells subcutaneously inoculated into BALB/c mice). Tumor lysates were subjected to western blotting for analysis of key inflammatory markers, ELISA for analysis of cytokines, and immunohistochemistry for analysis of inflammatory markers. Metabolite profiles of scallion extracts were analyzed by LC-MS/MS. Scallion extracts, particularly hot-water extract, orally fed to mice at 50 mg (dry weight)/kg body weight resulted in significant suppression of tumor growth and enhanced the survival rate of test mice. At the molecular level, scallion extracts inhibited the key inflammatory markers COX-2 and iNOS, and suppressed the expression of various cellular markers known to be involved in tumor apoptosis (apoptosis index), proliferation (cyclin D1 and c-Myc), angiogenesis (VEGF and HIF-1α), and tumor invasion (MMP-9 and ICAM-1) when compared with vehicle control-treated mice. Our findings may warrant further investigation of the use of common scallion as a chemopreventive dietary agent to lower the risk of colon cancer.

## Introduction

Colon cancer is a leading cause of cancer mortality in Western countries; it is also increasing in prevalence in Asia [1], [2]. It is well recognized that risk factors for colon cancer development include genetic and environmental factors, and unhealthy lifestyles. Environmental factors and food in particular have been epidemiologically shown to be closely associated with human colorectal cancer [3]. Currently, therapeutic approaches for human colorectal cancer include radiotherapy, chemotherapy and surgery, or a combination of these approaches; however, these strategies are still often not satisfactory due to significant side effects and dose-limiting toxicities.

Given that complete removal of the causative agents of cancer is most often not possible, effective measures for prevention of cancer is an area currently under active investigation [4]. Nutritional or chemo-prevention of cancers using various antimutagens and/or anticarcinogens present in dietary supplements, especially from food/spice plants has been repeatedly shown to offer various degrees of protection against cancers; and sometimes high efficacy has been shown [5]. Currently, more than 50% of pharmaceutical drugs are derived from natural plant products [6], [7]. Epidemiologic studies have shown that a diet high in fruits and vegetables (including spices) can effectively reduce the risks of certain cancers, especially those originating in the digestive tract [7]. Individuals who consume a variety of plant-derived foods such as fruits, vegetables, and soybeans were found to have a significantly lower incidence of cancer. A specific strategy advocating a “five-a-day” consumption of colorful fruits, spices and vegetables has been proposed for cancer prevention [8].

*Allium fistulosum* L. Alliaceae, commonly known as scallion or Welsh onion, is a perennial herb that is a member of the onion family. It is frequently used in a spectrum of cuisines worldwide, including those of Asia, Europe and America. *A. fistulosum* has also been employed in traditional Chinese medicine (TCM) to treat a variety of diseases. In spite of such popular usage, to date the number of experimental studies that have investigated the anti-cancer or other medicinal properties of *A. fistulosum* is very limited. *A. fistulosum* has been shown to scavenge free radicals and effectively inhibit the expression of inflammatory markers iNOS and COX-2 under different lipopolysaccharide-stimulated and reduced low-density lipoprotein (LDL) oxidation conditions [9], to modulate aortic vascular tone [10], and to confer antihypertensive effects on rats fed with diets high in fat and sucrose [11]. Recent studies showed that ethanolic extract of *A. fistulosum* significantly inhibited the mass of adipose tissue, fat accumulation and serum lipid concentrations through downregulation of the expression of key lipogenesis genes in the adipose tissue of high-fat diet-induced obese mice [12]. Furthermore, *A. fistulosum* has been shown to lower blood pressure and inhibit platelet aggregation [13], and exhibit antioxidant and anti-inflammatory activities [14].

Currently, little or no information is available about the possible antitumor effect of scallion extracts on colon or other types of cancers. The present study aimed to investigate the effect of various orally fed scallion crude extracts on apoptosis and other specific molecular markers for proliferation, angiogenesis and invasion of CT-26 colon tumors in treated mice and to evaluate possible tumor suppression mechanisms. The hot-water extract of scallion, at a low or modest oral dosage, was found to significantly induce apoptosis and downregulate a number of specific key molecular markers for tumor proliferation, inflammation, angiogenesis and invasion in test mice.

## Materials and Methods

### Preparation of Plant Extracts

Fresh scallion plants were grown and collected from specific farms in Yilang County, Taiwan. No specific working permits were required for the described field studies. Permission was obtained from collaborating farmers to use the scallion plants for the present study. The field studies did not involve any endangered or protected plant species. Authenticity of the plant species was verified, using anatomical and taxonomical phenotypes. The edible portions, i.e., the non-root, aerial-tissues of *A. fistulosum* were cut into small pieces for the preparation of extracts from leaves and stem. For the preparation of cold water extract, one-third of the *A. fistulosum* plant pieces were blended with cold double-distilled water (1∶5). The remaining pieces of *A. fistulosum* were divided into two parts, one part was boiled in water (1∶5) at 100°C for 1 h, and the other part was immersed in 100% ethanol (1∶5) for one week. After filtering through filter paper, the crude extracts were concentrated by lyophilization. The “dried extracts” were solubilized in 1% aqueous Tween 80 as a stock solution and further diluted in distilled water before feeding to test mice.

### Cell Line and Culture Conditions

The CT-26 murine colorectal carcinoma cell line was purchased from the American Type Culture Collection (ATCC) and maintained at 37°C in a humidified incubator in RPMI-1640 medium supplemented with 10% fetal bovine serum under 5% CO_2_. Cells were passaged twice a week by removing the adherent cells with trypsin/EDTA in phosphate buffered saline.

### Animals

Male BALB/c mice (National Laboratory Animal Center, Taipei, Taiwan) were given a standard laboratory diet and distilled water *ad libitum* and kept on a 12-h light/12-h dark cycle at 22±2°C. All animal studies for this research project were performed in strict accordance with the recommendations documented in the Guide for the Institutional Animal Care and Use Committee (IACUC) of Academia Sinica, Taiwan. The protocol was approved by the IACUC of Academia Sinica (Protocol ID: 10-09-072 ) ( File S1).

### Experimental Design

One week after transplantation of mouse colon tumor cells (CT**-**26), mice were randomly assigned to the following treatment groups (n = 10): (i) 1% Tween 80 (50 µl) as vehicle control (Control); (ii) Scallion cold-water extract (50 mg weight of dry/crude extract/kg body weight (b.w.) once daily, administered orally) (SCE-C); (iii) Scallion hot-water extract (50 mg/kg b.w. once daily, administered orally) (SCE-H); (iv) Scallion ethanolic extract (50 mg/kg b.w. once daily, administered orally) (SCE-E), and (v) curcumin (50 mg of pure compound/kg b.w. once daily, administered orally), as a positive control.

### *In vivo* Evaluation in CT-26 Mouse Tumor Models

Male BALB/c mice were implanted subcutaneously with cultured CT-26 cells (1×10^5^ cells/mouse). When tumor dimensions reached approximately 3 mm×3 mm, the animals were pair matched into treatment and control groups (day 7). Data were pooled from two independent experiments and each group consisted of 5 test mice that were ear-tagged and followed up individually throughout the study. Oral administration of scallion crude extracts or vehicle control into test mice was started on day 8. Each extract was administered at a dose of 50 mg/kg b.w./day. The control group received vehicle control (1% Tween 80). Mortality and body weight were monitored daily, and tumor size was measured on alternate days by calipers. The tumor volume was calculated using the following formula: tumor volume (mm^3^) = (length×width^2^)/2. On day 20, test mice were sacrificed by cervical dislocation, and tumors, livers, spleens and kidneys were quickly removed and weighed. Then the inhibition of rate of tumor was calculated. The tumor inhibition rate (%) = [(C − T)/C]×100, where C is the average tumor weight of the vehicle control group, and T is the average tumor weight of scallion crude extracts and curcumin treated groups. Another set of mice was sacrificed when they became moribund, and the date of death was recorded to calculate the survival time.

At the end of experiment, mice were sacrificed and tumors excised and weighed. One half of each tumor tissue sample was fixed with formalin and paraffin-embedded for immunohistochemistry staining, the other half snap-frozen in liquid nitrogen and stored at −80°C. Based on tumor growth data, selected tumor specimens from mice treated with specific scallion extracts and control groups were processed for analysis of specific cellular and molecular markers.

### Primary Culture of Colorectal Cells

Freshly excised colon tissues were obtained from 6–8 weeks old male BALB/c mice and flushed with sterilized PBS containing 100 units/ml penicillin, 100 µg/ml streptomycin (Invitrogen, USA) and 1x Antibiotic*-*Antimycotic (Gibco, USA) to remove soiling. The colon segment was opened up by longitudinal incision and soaked in 0.04% sodium hypochlorite in PBS for 15 min at room temperature to disinfect. Then colon tissues were minced into small pieces (1×2 mm) and digested for 2 hours with 1 mg/ml collagenase IV and 0.5 mg/ml hyaluronidase. The digested tissue mixture was filtered through nylon mesh to remove the debris. The isolated colorectal cells and their aggregates were harvested and cultured in DMEM/F12 medium supplemented with 10% FBS, 100 units/ml penicillin, 100 µg/ml streptomycin and 1×Antibiotic*-*Antimycotic.

The primary cultures of colorectal cells and CT-26 cells were seeded into 96-well plate for 24 hours. Test cells were then treated with SCE-C or SCE-H or SCE-E extract at different concentrations for 48 hours. After treatment, cell viability was measured by 3-(4,5-dimethylthiazol-2-yl)-2,5-diphenyl tetrazolium bromide (MTT)-based colorimetric assays.

### *In situ* Analysis of Apoptosis by TUNEL Assay

Apoptotic activities in tumor tissues were detected by terminal deoxynucleotide transferase (TdT)-mediated dUTP nick-end labeling (TUNEL) assay performed using the *In Situ* Cell Death Detection Kit (Roche, Mannheim, Germany) according to the manufacturer’s protocol. The degree of apoptosis was evaluated by counting the positive cells (brown color-stained) present in 10 randomly picked microscopic fields (400× magnification) for each tumor tissue sample. Representative images on positive staining of tumor tissues are shown, and quantification data is shown as a bar diagram.

### Immunohistochemistry Staining of Biomarkers

At the termination of experiments, tumor tissues were removed for immunohistochemical studies. Briefly, tumor tissues embedded in paraffin blocks were microtome cut into 4 µm sections, deparaffinized and treated with citrate buffer (pH 6.0). They were then treated with blocking buffer for 60 min. The processed tissue sections were then incubated with specific antibodies against COX-2, cyclin D1, VEGF and MMP-9 (Cell Signaling Technology, MA) overnight at 4°C. Subsequently, slides were treated for 1 h with secondary, goat anti-rabbit antibodies conjugated with horseradish peroxidase (Santa Cruz Biotechnology, CA), and developed with 3,3′-diaminobenzidine (BD Biosciences, CA). Finally, tissue sections were rinsed in distilled water, counterstained with Mayer’s hematoxylin and mounted with DPX mounting medium for microscopic examination. Immunohistograms were taken with an Olympus DP-70 camera on a Nikon Eclipse E800 microscope. Immunohistochemical staining of tissue sections were performed in triplicate for each antibody.

### Quantification of Immunostaining Activity in Tumors

Cells that were immunohistochemically positive for inflammatory molecules were recorded by counting the brown color-stained cells in 10 randomly selected fields at 400× magnifications. In other cases, immunoreactivity (represented by the intensity of brown staining) was scored as 0 (no staining), +1 (very weak), +2 (weak), +3 (moderate) and +4 (strong staining). Representative images of positive staining (400×) of tumor tissues are shown and quantification of positive staining score was shown as a bar diagram.

### Preparation of Tumor Homogenates and Western Immunoblot Analysis

Two representative tumors were selected based on tumor data from each of the control (untreated) and treated groups, homogenized in non-denaturing tissue lysis buffer, and centrifuged at 14,000×g for 15 min. Protein concentration was measured by the Bradford assay, with bovine serum albumin as the standard. Equal amounts of protein per lysate were subsequently resolved on a SDS-polyacrylamide gel, transferred onto a PVDF membrane, and blocked for 1 h with 5% nonfat dry milk. Membranes were then incubated with the appropriate primary antibodies (Cell Signaling Technology, MA) overnight at 4°C and then with the appropriate HRP-conjugated secondary antibodies (Santa Cruz Biotechnology, CA). Blots were scanned with Adobe Photoshop 6.0 with minimum background. The membranes probed with anti–β-actin antibody were used as a sample-loading control. Enhanced chemiluminescence reagents were used to detect the signals following the manufacturer’s instructions. Immunoblot analyses were conducted in duplicate for each antibody.

### Overexpression of Transgenic VEGF in CT-26 Cells

The vascular endothelial growth factor (VEGF) expression plasmid pBLAST49-mVEGF was purchased from Invivogen (San Diego, CA, USA), and was used to transfect into CT-26 cells using Turbofect Transfection reagent (Fermentas, USA). In brief, 3×10^5^ CT-26 cells were seeded into 6-well plate and grew for 24 hours. For transfection, 4 µg pBLAST49-mVEGF plasmid was diluted in 400 µl serum-free RPMI-1640 medium, and 6 µl Turbofect reagent was added to the diluted plasmid solution. The Turbofect/plasmid mixture was incubated at room temperature for 20 min. Aliquots of 400 µl of plasmid mixtures were added into each well and incubated with CT-26 cells for 24 hours. After transfection, cells were harvested and seeded into 96-well plate for 24 hours, and treated with SCE-C or SCE-H or SCE-E extract at 100 µg/ml concentration for another 48 hours. After treatment, the cell viability was examined by MTT assay.

### ELISA Analysis of Inflammatory Cytokines

The levels of IL-6 and TNF-α in tumor cell lysates were measured using an enzyme linked immunosorbent assay (ELISA) kit (R&D systems, Minneapolis, MN) according to the manufacturer’s protocol. All assays were performed in triplicate.

### LC-ESI-Q-TOF MS Analysis

For LC-MS analysis, acetonitrile (ACN) with 0.1% formic acid (FA) and water with 0.1% formic acid (LC-MS grade, J. T. Baker, Phillipsburg, NJ) were used for separation in the mobile phase. Sulfadimethoxine and formic acid were purchased from Fluka (Steinheim, Germany). LC-MS was performed with a LC system (ACQUITY UPLC, Waters, Millford, MA) coupled to a hybrid Q-TOF mass spectrometer (Synapt HDMS, Waters, Manchester, UK). Test samples were separated online with a reverse-phase column, (HSS T3 C18, 1.8 µm, 2.1 mm×150 mm, Waters, Milford, MA), which was kept in a column oven at 40°C. The mobile phases for positive ion mode consisted of 0.1% formic acid in 2% ACN (buffer A) and 0.1% formic acid in 100% ACN (buffer B). The mobile phases for negative ion mode consisted of 2% ACN (buffer A) and pure 100% ACN (buffer B). The sample injection volume was 10 µL and the mobile phase flow rate was 400 µl/min using a 4 min gradient from 5–95% acetonitrile/water. The mass spectrometer equipped with a lock electrospray ionization probe was operated in both positive and negative (ESI) mode. The electrospray voltage was set to 3 kV for positive ion mode and −2.5 kV for negative ion mode, and the cone voltage was 40 V. The cone and desolvation gas flow rates were 50 and 700 µL/h, respectively. A lock mass calibration of sulfadimethoxin (0.5 mg/L) in water/MeOH (50∶50 v/v) was introduced by the HPLC pump (LC-10ATVP, Shimadzu, Japan) and split to the lockspray probe with a flow rate of 5 µL/min. The acquisition method was set to one full MS scan (50–990 m/z) with 0.2 sec scan time in centroid data mode.

### Processing of Data Files

The LC-MS data were analyzed by MarkerLynx XS version 4.1 SCN639 (Waters, Milford, MA). In this application, the peaks in the 0.75–5.75 min range of LC-MS data were detected and the noise was reduced in both of the LC and MS domains. The extracted peak information was then processed to remove the peak-to-peak noise, deisotope and filter the MS peaks with the intensity lower than 50 counts. A list of the intensities of the processed peaks for each of the LC-MS data was generated using retention time (RT) and m/z data pairs as the identifier of each peak. The processed data for each test sample were combined and aligned for each of the RT-m/z pairs to generate the final data table. The ion intensities for each peak were then normalized within each sample and the 3-dimensional data, peak identifier (RT-m/z pair), sample name, and ion intensity were analyzed by principle component analysis (PCA).

### Statistical Analysis

Data are presented as mean ± SD. To assess statistical significance, values were compared to controls using the student t-test by using the Prism GraphPad 5 software. *P<0.05; **P<0.01; ***P<0.001 were considered statistically significant.

## Results

### Specific Scallion Extracts Suppress Colon Tumor Growth and Increase Survival Rate in Test Mice

**Figure 1:**
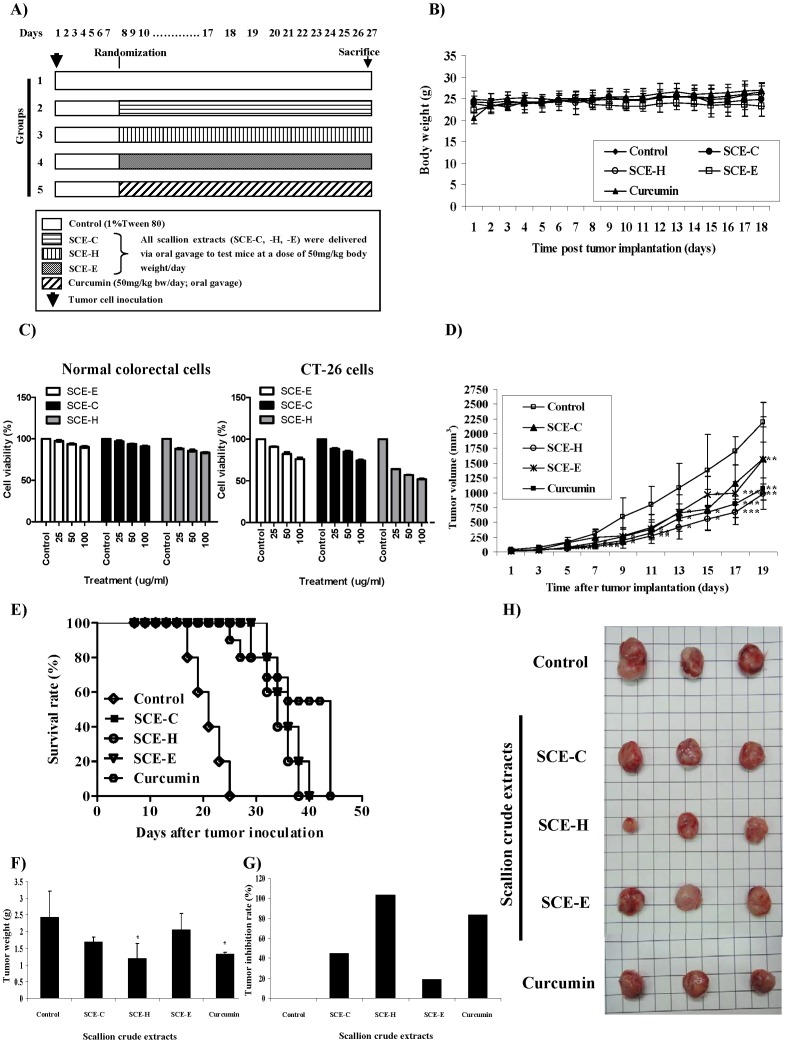
Effect of three different scallion extract preparations on CT-26 tumor-bearing mice. (A) Schematic diagram of the experimental design for the in vivo antitumor effect of scallion extracts. (B) Mean body weight. (C) The differential cytotoxic effect of SCE treatments on colon tumor and normal colorectal cells. (D) Final tumor volume. (E) Survival rate of test mice. (F) Weight of excised tumors. (G) Tumor inhibition rate. (H) Representative photographs of tumors from tumor-bearing mice of all test groups.

To evaluate possible antitumor effects of scallion crude extracts on the growth of CT-26 colon tumors in BALB/c mice, test animals were treated daily with scallion crude extracts at a dosage of 50 mg/kg body weight/day. A schema of our experimental design is shown in Figure 1A. Oral gavage delivery of hot (SCE-H) and cold (SCE-C) water extracts, and ethanol extract (SCE-E) of scallion had little or no effect on body weight of test mice (Figure 1B). Furthermore, we did not observe any adverse effects of treatment as monitored by the activity and posture of experimental mice. In addition, the weights of liver, kidney, heart, spleen and lung were not significantly different between the control and scallion extract-treated CT-26 tumor-bearing mice (data not shown). In vitro tests also demonstrated that the test scallion extracts, especially the SCE-H extract, were far less cytotoxic to the primary cultures of normal colorectal cells than to the malignant/cancerous CT-26 cells in vitro (Figure 1C).

The hot-water extract (SCE-H) inhibited tumor growth more effectively than the cold-water or ethanolic extracts (SCE-C, SCE-E); however, all the crude extracts tested conferred an inhibitory effect in comparison with the vehicle control (Figure 1D, 1E). Tumor volumes and the survival rate of tumor-bearing mice showed that the suppression of tumor growth in the SCE-H treated group was more significant (***P<0.001) when compared with the control group. SCE-H (50 mg/kg b.w. per day) was almost as effective as the same dosage of pure curcumin compound (50 mg/kg/b.w./day), which was used as a positive control, at suppressing tumor growth and associated mechanisms. Curcumin (diferulomethane), a natural product of the spice turmeric (*Curcuma longa*), has been shown to exhibit chemopreventive activity against various types of cancers [15]–[17] and is known to be nontoxic to humans at high dosages (e.g., 8 g/day) [18]. The cold-water and ethanolic extracts of scallion, on the other hand, were considerably less effective in suppressing growth of CT-26 mouse colon tumors (Figure 1D).

Effects of scallion extracts on suppression of tumor growth were then assessed by tumor weight (Figure 1F), tumor inhibition rate (Figure 1G) and tumor morphology (Figure 1H) in comparison with tumors in control group mice. Tumor weight on day 20 was significantly (*P<0.05) lower in the group administered an oral dose of hot-water (SCE-H) extract daily compared with that of the control mice. In addition, an increased rate of tumor inhibition was observed in the SCE-H-treated group when compared to the control group and the other treatment groups.

### Hot Water Scallion Crude Extract (SCE-H) Induces Apoptosis and Inhibits Cell Proliferation

**Figure 2:**
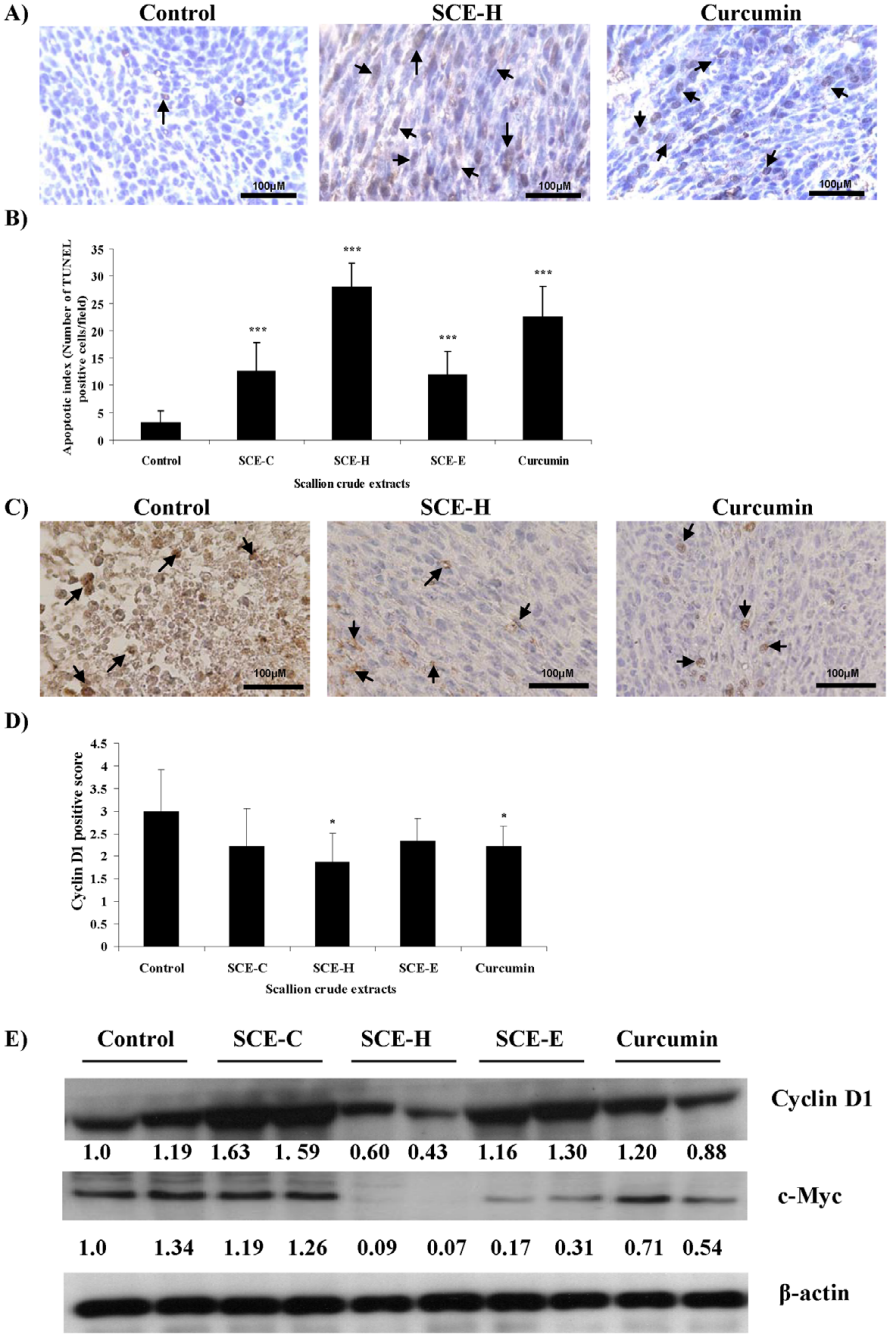
Pro-apoptotic and anti-proliferative effects of scallion extracts on growth of CT-26 tumor cells in vivo. At the end of the experiment a portion of each test tumor from the control and scallion extract-treated groups was collected and subjected to TUNEL assay and immunohistochemical analysis of cyclin D1 expression. Representative images of TUNEL staining (A), and cyclin D1 (C) immunoreactivity are shown (400× magnification). Quantification of apoptotic index and proliferation are shown as number of TUNEL-positive cells (B) and positive scores of cyclin D1-expressing cells (D), respectively. A portion of two selected tumors from each test group were analyzed for cyclin D1 and c-Myc protein expression by western blotting. Densitometric data are shown as fold-change versus the control under each band (E).

The *in vivo* apoptosis-inducing and antiproliferative effects of scallion crude extracts were examined by analyzing the levels of key molecular markers: TUNEL staining was used to evaluate tumor cell apoptosis and the level of cyclin D1, and immunohistochemical detection and immunoblots of c-Myc were used to evaluate tumor cell proliferation (Figure 2A–E). Quantification of TUNEL-positive cells showed that the SCE-H group had a more significantly (***P<0.001) increased apoptotic index than the other treatment groups and the control mice (Figure 2B). Overexpression of cyclin D1 is known to be associated with enhanced cell proliferation. The number of cyclin D1-positive cells found in the tumors of the control mice were significantly reduced in mice treated with SCE-H (Figure 2C, 2D). The immunohistochemistry results (Figure 2C) were further supported by immunoblot analysis (Figure 2E) of two randomly selected tumor samples from each of the tested groups, which showed a similar expression pattern in response to treatment with SCE-H. Interestingly, SCE-C and SCE-E seemed to have slightly increased expression of cyclin D1 (Figure 2E). It is important to note here that Western blot analysis may sometimes not accurately reflect the effect on test tumor cells at the cellular or tissue level. The inconsistency between the data shown in Figure 2D and 2E might be partly due to the effect of test phytoextracts on different cell types present in specific tumor microenvironment, e.g., the “heterogenous tumor stromal tissues” as partially reflected by different tissue colors in Figure 1H. None the less, our results (Figure 2A–E) together demonstrate that the scallion hot-water extract (SCE-H) can consistently and effectively confer pro-apototic as well as anti-proliferative effects in test colon tumor tissues.

### SCE-H Effectively Decreases Expression of Inflammatory Molecular Markers

**Figure 3:**
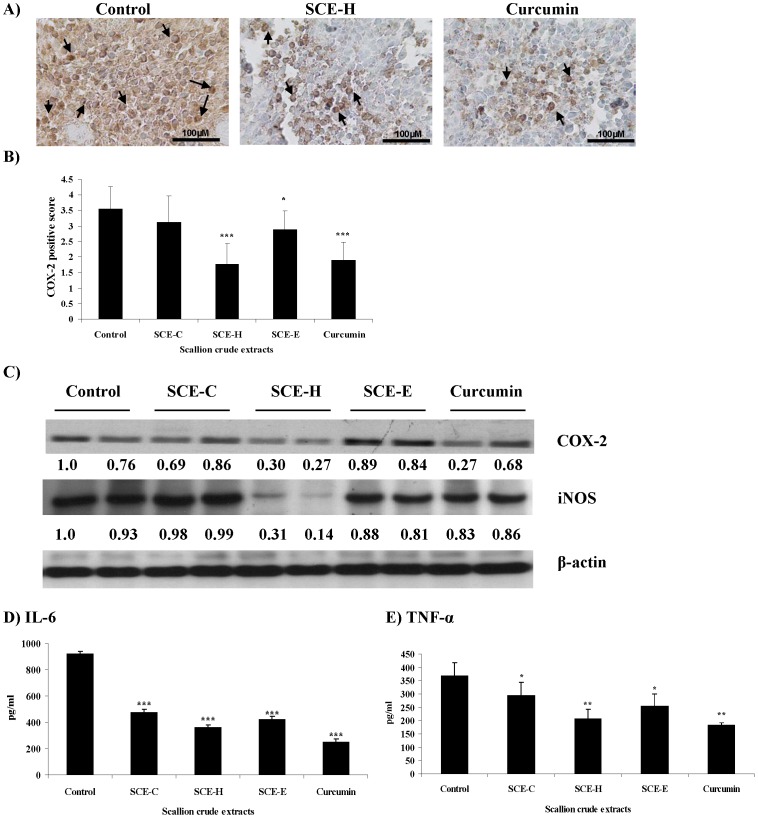
Effect of scallion extracts on expression of iNOS, COX2 and cytokines in test mice. At the end of the experiment portions of each tumor from the control and scallion extract-treated groups were collected and subjected to immunohistochemical and western blot analyses of test marker proteins. (A) Representative images (400× magnification) of immunohistochemical staining of COX2 expression. (B) Quantification of COX2-expressing cells as positive scores. (C) Western blot analysis of iNOS and COX2 protein expression in two independent tumors from each test group. (D) Levels of IL-6 and (E) TNF-α in tumor lysates analyzed by ELISA.

Possible roles of the specific inflammatory molecules COX-2 and iNOS in colon cancer have been documented, and these molecular markers can be modulated by chemopreventive and anti-inflammatory agents [19]. Increased expression of COX-2 and iNOS was observed in the tumor-bearing control group mice as compared to normal, non tumor-bearing mice (data not shown). After treatment with SCE-H, the expression of COX-2 was significantly reduced, as observed by immunohistochemical (Figure 3A, 3B) as well as immunoblot analyses (Figure 3C). Quantification of COX-2 staining based on the intensity of immunoreactivity in test cells also showed higher levels of expression of COX-2 in the control group than in the scallion crude treatment groups (Figure 3B). It is also intriguing to observe that, whereas SCE-H strongly suppressed both COX-2 and iNOS activities in test tumors, curcumin only suppressed COX-2 activity and not iNOS activity in comparison with the control group (Figure 3C). Overall, SCE-H, which as a crude extract contains a spectrum of phytochemicals, was similarly or even more potent in suppression of COX-2 and iNOS activities in test tumors than curcumin (Figure 3B, 3C). In contrast, SCE-C and SCE-E extracts conferred a much less pronounced effect on both inflammatory markers tested (Figure 3B, 3C). This differential effect may warrant future evaluation. As expected, treatment with SCE-H significantly decreased levels of pro-inflammatory cytokines IL-6 and TNF-α in comparison with the high levels of IL-6 and TNF-α detected in control mice (Figure 3D, 3E). Significant effects were also detected in mice treated with SCE-C and SCE-E, although the reductions in expression of IL-6 and TNF-α were smaller than those observed for treatment with SCE-H and curcumin.

### SCE-H Inhibits the Expression of Angiogenesis Markers

**Figure 4:**
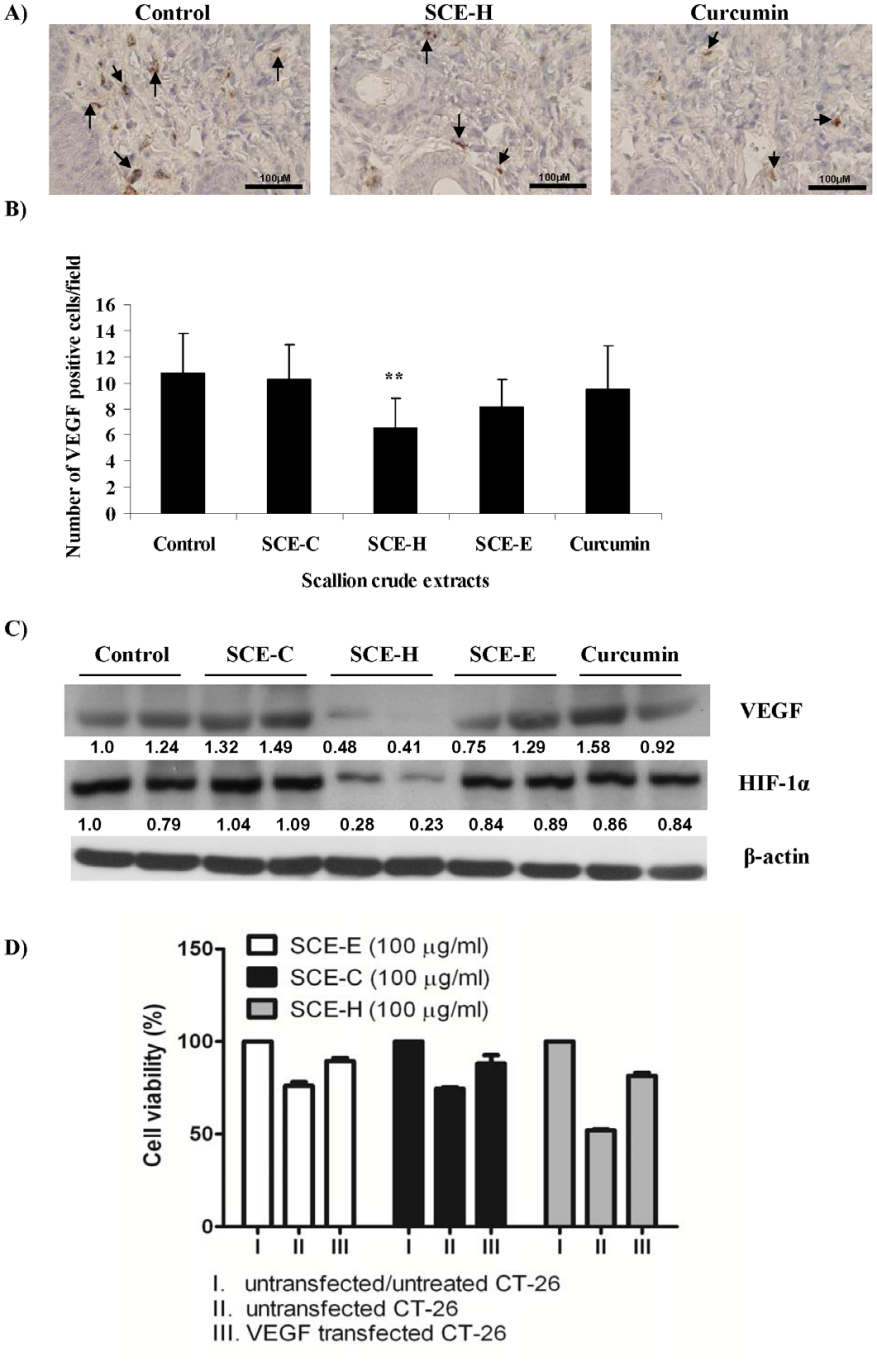
Effect of scallion extracts on expression of VEGF and HIF-1α in CT-26 tumor-bearing mice. At the end of the experiment portions of each tumor from the control and scallion extract-treated groups were collected and subjected to immunohistochemical, western blot and marke**r** protein analyses. (A) Representative images of immunohistochemical staining of VEGF (400× magnification). (B) Quantification of VEGF-expressing cells shown as number of positive cells. (C) A portion of two selected tumors from each group were analyzed for VEGF and HIF-1α protein expression using western blotting. (D) Overexpression of transgenic VEGF effectively rescued the cell viability of test CT-26 cells when treated with SCE extracts.

Vascular endothelial growth factor (VEGF) is a potent and specific angiogenic factor of tumor-induced angiogenesis. In our study, immunohistochemical analysis of tumors from the control group showed clear immunoreactivity for VEGF. As shown in Figure 4A, decreased immunoreactivity was observed in mice treated with SCE-H. Quantification of the immunohistochemical staining showed that VEGF was more significantly decreased in the SCE-H treatment group (**P<0.01) than in the SCE-C, SCE-E and curcumin-treated groups, compared to the control group (Figure 4B). Results obtained by immunoblot analysis were consistent with the immunohistochemical data (Figure 4C). To further investigate the involvement of VEGF in response to SCE-H, we transfected CT-26 cells with a VEGF gene expression plasmid and subjected test cells to treatment with scallion extracts. As compared to control cells, overexpression of transgenic VEGF effectively “rescued” the cell viability of transfected CT-26 cells that were treated with SCE-H extracts, resulting in an increase from 51% to 81% (Figure 4D). This reversal effect strongly suggests that VEGF is one of the molecular targets in test colon tumors that the specific phytochemicals present in SCE-H can act upon. In addition to VEGF, we observed that the level of HIF-1α protein was also drastically reduced in the SCE-H treatment group, in stark contrast to other treatment groups (Figure 4C).

### SCE-H Downregulates Expression of MMP-9 and ICAM-1

**Figure 5:**
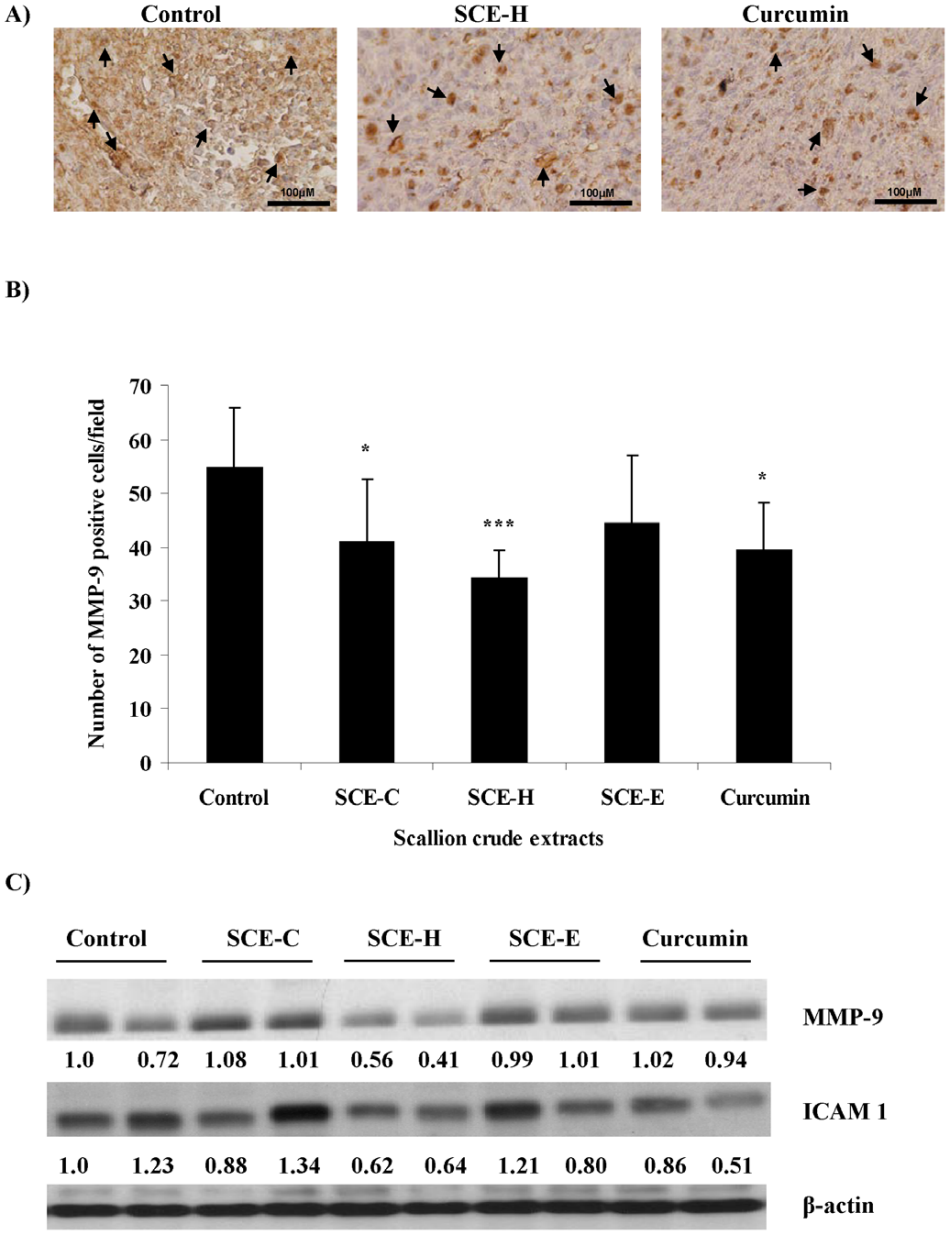
Effect of scallion extracts on MMP9 and ICAM-1 expression in CT-26 tumor-bearing mice. Immunohistochemical staining and western blot analyses for MMP9 and ICAM-1 are performed as described above in Fig. 4. (A) Representative images of immunohistochemical staining of MMP9 expression (400× magnification). (B) Quantification of MMP9-expressing cells shown as number of positive cells. (C) A portion of two selected tumors from each group were analyzed for MMP9 and ICAM-1 protein expression using western blotting.

Immunohistochemical analysis subsequently showed that MMP-9 expression in CT-26 tumors of test mice was significantly inhibited by treatment with SCE-H (Figure 5A, 5B). We further confirmed this effect of SCE-H by immunoblot analysis. Western blot analysis (Figure 5C) showed that treatment with SCE-H effectively reduced the levels of MMP-9 and ICAM-1 to approximately 50% of the untreated (control) levels. Curcumin had a similar or slightly lower effect. The effects from treatment with SCE-C and SCE-E were again not obvious or consistent. In addition, SCE-C appeared to have slightly increased expression of MMP-9, as measured by Western blotting (Figure 5C). This result seems not to correlate with the immunohistochemical data shown in Figure 5A and 5B. As mentioned above, we consider this difference between the Western blotting and immunohistochemistry results may reflect the possible effect of SCE-C and SCE-E on varying stromal cell types present in specific tumor microenvironments. Future studies are needed to address such a possibility.

### Metabolite Profiling and Candidate Active Phytochemical Components in Scallion Extracts

**Figure 6:**
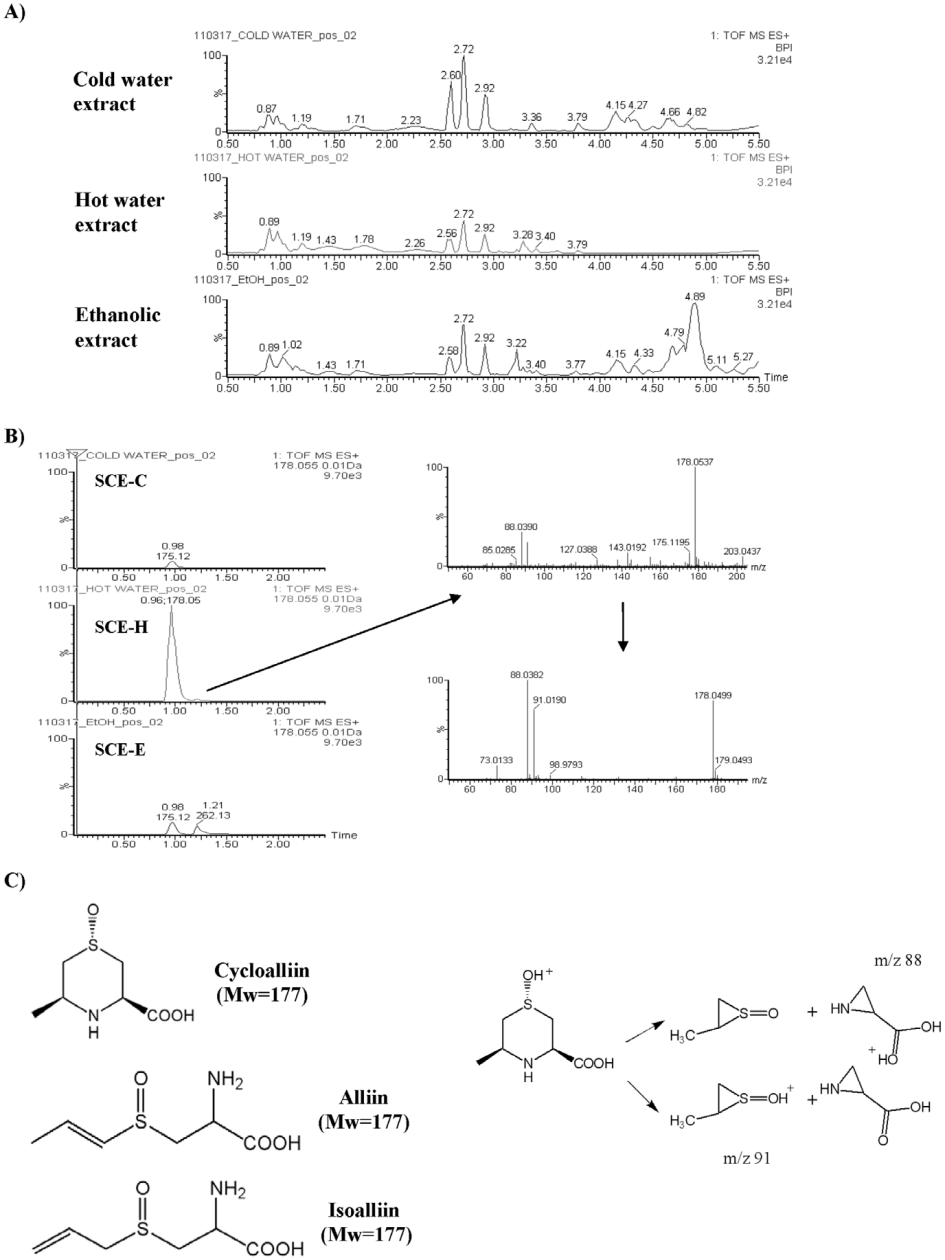
LC-MS/MS metabolite profiling of different scallion plant extracts. (A) LC-MS profiling of extractable plant metabolites. (B) MS/MS analysis of specific peaks from scallion hot water extract. (C) Chemical structures of cycloalliin isomers and the proposed mass fragmentation of cycloalliin.

Liquid chromatography coupled with mass spectroscopy (LC-MS) and principal component analysis (PCA) analyses were used to evaluate the differences and similarities in metabolite profiles and to identify the key phytochemicals present in different scallion extract preparations. Scallion tissue extracts prepared by different extraction methods displayed readily distinguishable metabolite profiles as revealed by LC-MS (Figure 6A). Furthermore, LC-MS/MS analysis of the metabolites present in SCH-H exhibited a high level unique mass peak (m/z 178), and this peak was presented at a very low level or absent in SCH-C and SCH-E. Chemical data based analyses indicate that this peak may represent alliin isomers, including alliin, isoalliin and cycloalliin. Further elucidation of the mass fragments by LC-MS/MS analysis, as shown in Figure 6B and 6C, determined that the sulfur-containing phytochemical cycloalliin, not alliin or isoalliin, was the most likely secondary metabolite that was present at a significantly higher level in SCE-H than in the SCE-C and SCE-E extracts of scallion. Interestingly, cycloalliin has been previously identified from garlic bulb, and reported to confer certain anti-tumor activities [20]. Other bioactivity-related mass spectrum peaks were also detected, and they are currently being systematically analyzed to identify the single chemical compounds.

## Discussion

Dietary chemoprevention of cancer has gained increasing interest in both developed and developing countries as a potentially important concept in public health. Plant materials such as vegetables, fruits and spices have been extensively studied for their chemoprotective properties, and many have been suggested to confer antioxidant, anti-inflammatory, and antitumor or cancer chemoprevention activities [21], [22]. Recent findings [22], [23] also show that various immunomodulatory activities, including those of different cytokines, chemokines and immune cell types are strongly linked to various malignancies such as colon and liver cancers [24]. In the present study, we investigated the effect of oral administration of a hot-water extract of scallion (SCE-H) on colon tumors in a mouse CT-26 tumor model. We demonstrated that SCE-H can markedly delay tumor incidence and reduce the growth of CT-26 inoculated tumors in BALB/c mice. Mechanistically, SCE-H inhibited cellular activities pertaining to proliferation, inflammation and angiogenesis, resulting in increased apoptosis and decreased expression of specific key inflammation and angiogenesis markers. These findings suggest further investigation of the effect of scallion extract on other animal models for colon cancer such as chemically-induced and transgenic mouse tumors is warranted.

**Figure 7:**
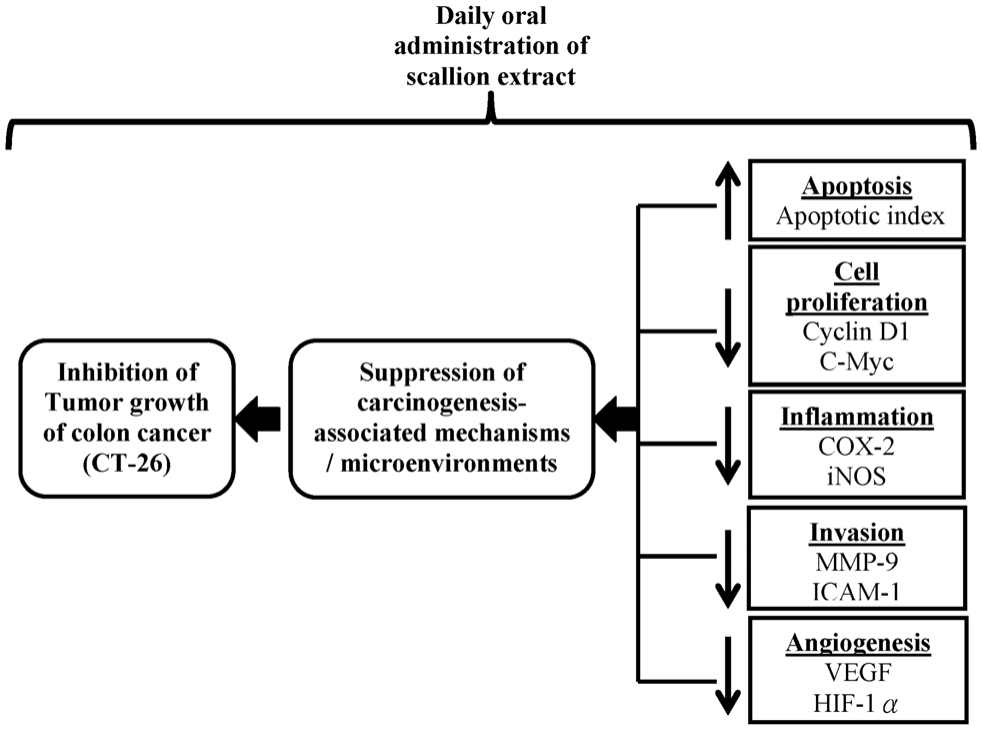
Scallion hot water extract may act by targeting multiple signaling pathways. Schematic representation of proposed anti-cancer mechanisms for the inhibition of colorectal tumor growth by dietary uptake of Allium fistulosum hot water extract.

In recent years a number of studies have suggested that targeting a single cellular or physiological event alone is not sufficient to control cancer growth; accordingly, agents that can target multiple tumorigenic events and associated mechanisms or tumor-associated microenvironments may be more effective in controlling cancer growth and progression [21], [25]. Consistent with this hypothesis, in our present study, SCE-H showed a strong chemopreventive efficacy in CT-26 tumor cell-inoculated mice, seemingly by inducing apoptosis, and inhibiting tumor-cell proliferation, inflammation and angiogenesis (Figures 2, 3, 4, 5, Figure 7).

Colon carcinogenesis is a multistage process, and enhanced cell proliferation and ablation of apoptosis are early events for its progression [26]. Apoptosis and cell proliferation biomarkers are commonly used to investigate the efficacy of potential chemopreventive agents [27], [28]. In this study, we evaluated specific biomarkers for apoptosis and cell proliferation such as cyclin D1 and c-Myc (Figure 2E). Cyclin D1 is known to play a crucial role in the development of specific subtypes of human cancers and it can promote progression through the G1-S phase of the cell cycle [29]. Our results may suggest that the inhibition of tumor-cell proliferation observed with SCE-H may be associated with G1-phase cell-cycle arrest. The transcription factor c-Myc is known to be overexpressed in different types of cancers including colon cancer [30]. Our present study shows that c-Myc expression was effectively downregulated by treatment of test mice with SCE-H. The apoptosis index (Figure 2A, 2B) was increased and the expression of key proliferation markers cyclin D1 and c-Myc were downregulated in test colon tumors, providing good *in vivo* molecular evidence for the antiproliferative efficacy and associated antitumor activities.

Inflammatory markers iNOS and COX-2 are useful prognostic markers of colorectal cancer [31]–[33]. Nitric oxide (NO), produced from iNOS mediates inflammation, influences tumor formation by promotion of various mechanisms in the colon [33], suppresses DNA repair enzyme activities, and inhibits apoptosis through nitrosylation of caspases in colorectal cancer [34]. We show in our present study that the SCE-H treatment can effectively downregulate the expression of iNOS (Figure 3C), suggesting that SCE-H may confer anti-inflammatory activity through the suppression of proinflammatory mediators. COX-2 is overexpressed in many cancers including colon cancer [35] and, as expected, CT-26 tumors in our test mice were indeed detected to contain high levels of COX-2; in contrast, mice treated with SCE-H showed a significant decrease in the level of COX-2 (Figure 3B, 3C) in tumor tissues. Scallion hot-water extract also effectively reduced the levels of pro-inflammatory cytokines IL-6 and TNF-α in mice bearing CT-26 tumors (Figure 3D, 3E), thus demonstrating strong anti-inflammatory activity. We therefore suggest that SCE-H may confer its anti-tumor activity via inhibition of inflammation-associated carcinogenesis or inhibition of inflammation in the tumor-associated microenvironment [36].

Many chemopreventive agents can downregulate expression of vascular endothelial growth factor (VEGF), a hallmark of tumor angiogenesis [37]–[39]. Hypoxia is a major pathophysiological condition that regulates angiogenesis. A specific transcription factor, hypoxia-inducible factor 1 (HIF-1) has recently become a specific target for cancer therapeutics [40]–[42]. Our data shown in Figure 4B and 4C may indicate that HIF-1α-mediated upregulation of VEGF in test colon tumors may be targeted by phytochemicals present in the scallion hot-water extract, as this SCE-H extract concomitantly decreased the expression of HIF-1α and VEGF.

MMP-9 has been suggested to be a useful diagnostic marker for colon carcinogenesis [43]. ICAM-1 is constitutively expressed at low levels on the surface of various cell types; it is cytokine-inducible and has been implicated in tumor prognosis, progression, and metastasis of cancer [44]–[49]. In the present study, downregulation of expression of both MMP-9 and ICAM-1 were detected in tumors of mice treated with SCE-H (Figure 5C). Therefore, SCE-H may help inhibit tumor invasion or progression via MMP-9 and/or ICAM-1.

The minor differences in data for expression of cyclin D1 and MMP-9 between Western blotting and immunohistochemistry staining assays (the SCE-C and SCE-E groups in Figure 2 and 5) may be due to bona fide differential effects of the phytochemicals present in the three different plant extract preparations. As seen in Figure 6A, 6B and 6C, these three different phytoextracts did indeed contain different metabolite profiles, as revealed by LC-MS and MS/MS analyses. Different but overlapping candidate active principles may therefore contribute to the differences in detected bioactivities (e.g., cyclin D1 expression seen in Figure 2D and 2E). It has been widely speculated that phytoextracts in traditional herbal medicine formulations, when prepared by different extraction methods, may exert differential effect in bioactivity. For example, recently it was reported that Artemisinin, a key phytochemical present in a traditional medicinal herb, is not stable in hot water extract, but much more stable in cold water extract [50]. In our study, we may have stumbled upon an opposite case. On the other hand different secondary plant metabolites, obtained from different extraction protocols (e.g., use of hot water, cold water or ethanol as solvent), may confer different bioactivities (e.g., via actions from different/separate molecular signaling systems), and exhibit overlapping but not identical anti-tumor or anti-inflammatory activities. We do not know at present time which one of the above possibilities is responsible for the difference in bio-activities we observed for SCE-H versus SCE-C and SCE-E, but we believe these possibilities may warrant future investigation. What we did show clearly in this study is that the SCE-H preparation confers the most potent and most consistent anti-tumor activities. The possible molecular mechanisms responsible for the mode of action of SCE-H, as described in Figure 7, were also specifically demonstrable by both Western blot and immunohistochemical analyses of key signaling pathways known to be involved in anti-inflammatory and anti-tumor bioactivities.

LC-MS/MS analysis of different scallion extract preparations revealed that isomers of alliin, specifically cycloalliin, are differentially present at higher concentrations in the scallion hot water extract than in the cold water or ethanolic extracts (Figure 6B, 6C). Previous reports [20], [51] have shown that alliin and its isomers including cycloalliin can confer readily detectable biological effects at the cellular level, including anti-tumor, anti-inflammatory and anti-microbial activities. Our present results demonstrate that SCE-H can effectively suppress colon tumor growth in test mice. We suggest that heat-stable phytochemicals such as the alliin isomers that are present at relatively abundant levels in scallion plants may contribute to the activity of this extract. Further studies are needed to confirm that these alliin isomers including isoalliin and cycloalliin can specifically confer the high anti-tumor potency that we detected in this study.

It is noteworthy that in this study the most highly efficacious dose of SCE-H, orally fed to test mice at 50 mg/kg body weight, would be equivalent to only 7.5 g fresh plant weight/70 kg human body weight/day when translated into human oral application [52]. This amount of scallion plant material is equivalent to only one piece of a medium size scallion plant, and this amount should be highly acceptable as a daily dietary uptake within both Asian and Western food/meal cultures.

In summary, the present findings provide a series of *in vivo,* co-relatable evidence that scallion hot-water extract, but not the cold-water or ethanolic extracts, has pro-apoptotic, antiproliferative, and antiangiogenic effects without detectable undesirable effects. The phytochemical constituents of the scallion hot-water extract, when orally administered, suppressed key inflammatory markers and inhibited various molecular and cellular markers involved in apoptosis, proliferation and angiogenesis, suggesting a mode of action targeting multiple signaling pathways *in vivo*, as is proposed in Figure 7. We therefore suggest that scallion plant materials and their extracts may have the potential to be systematically developed as a chemopreventive agent(s) or medicinal food against specific colon cancers. Furthermore, specific and improved methods of cooking of scallion plants may also provide a useful healthcare measures in the development of functionally verified, dietary supplements against colon cancers.

## Supporting Information

File S1 **Approval letter.** This is to certify that the animal protocol by the following applicant has been evaluated and approved by the Institutional Animal Care and Use Committee of Academia Sinica (AS IACUC).
(PDF)
