# Supplementary material for: Dietary Administration of Scallion Extract Effectively Inhibits Colorectal Tumor Growth: Cellular and Molecular Mechanisms in Mice
Source: PLoS One. 2012 Sep 14;7(9):e44658. doi: 10.1371/journal.pone.0044658 (PMC3443092; doi:10.1371/journal.pone.0044658)
Supplement: File S1 — Approval letter. This is to certify that the animal protocol by the following applicant has been evaluated and approved by the Institutional Animal Care and Use Committee of Academia Sinica (AS IACUC). (PDF) [file pone.0044658.s001.pdf]

From: iacuc@gate.sinica.edu.tw

Sent Date: 星期一, 九月 05, 2011 10:05:23 上午

To: nsyang@gate.sinica.edu.tw

Cc:

Bcc:

Subject: IACUC Protocol Approved: 10-09-072, 楊, 寧蓀

Message:

IACUC has approved the protocol with the following details.

Protocol ID: 10-09-072
Principal Investigator: 楊, 寧蓀
Department: 農生中心(ABRC)
Protocol Title: 應用食材佐料或藥用植物以抗發炎及抗高血壓之營養 /功能性代謝體之研究
Use of Specific Spice Plants in Diet for Anti-Inflammation and Anti-Hypertension Activities: Nutritional and Functional Metabolomics Approaches
Review Type: FULLBOARD
Approval Date: 05/09/2011
